# A Gene-Based Linkage Map for *Bicyclus anynana* Butterflies Allows for a Comprehensive Analysis of Synteny with the Lepidopteran Reference Genome

**DOI:** 10.1371/journal.pgen.1000366

**Published:** 2009-02-06

**Authors:** Patrícia Beldade, Suzanne V. Saenko, Nicolien Pul, Anthony D. Long

**Affiliations:** 1 Institute of Biology, Leiden University, Leiden, The Netherlands; 2 Instituto Gulbenkian de Ciência, Oeiras, Portugal; 3 Department of Ecology and Evolutionary Biology, University of California Irvine, Irvine, California, United States of America; University of Arizona, United States of America

## Abstract

Lepidopterans (butterflies and moths) are a rich and diverse order of insects, which, despite their economic impact and unusual biological properties, are relatively underrepresented in terms of genomic resources. The genome of the silkworm *Bombyx mori* has been fully sequenced, but comparative lepidopteran genomics has been hampered by the scarcity of information for other species. This is especially striking for butterflies, even though they have diverse and derived phenotypes (such as color vision and wing color patterns) and are considered prime models for the evolutionary and developmental analysis of ecologically relevant, complex traits. We focus on *Bicyclus anynana* butterflies, a laboratory system for studying the diversification of novelties and serially repeated traits. With a panel of 12 small families and a biphasic mapping approach, we first assigned 508 expressed genes to segregation groups and then ordered 297 of them within individual linkage groups. We also coarsely mapped seven color pattern loci. This is the richest gene-based map available for any butterfly species and allowed for a broad-coverage analysis of synteny with the lepidopteran reference genome. Based on 462 pairs of mapped orthologous markers in *Bi. anynana* and *Bo. mori*, we observed strong conservation of gene assignment to chromosomes, but also evidence for numerous large- and small-scale chromosomal rearrangements. With gene collections growing for a variety of target organisms, the ability to place those genes in their proper genomic context is paramount. Methods to map expressed genes and to compare maps with relevant model systems are crucial to extend genomic-level analysis outside classical model species. Maps with gene-based markers are useful for comparative genomics and to resolve mapped genomic regions to a tractable number of candidate genes, especially if there is synteny with related model species. This is discussed in relation to the identification of the loci contributing to color pattern evolution in butterflies.

## Author Summary

Butterflies and moths (called the Lepidoptera) are a large and diverse group of insects that has long captured the attention of biologists and laymen. The colorful patterns on the wings of butterflies, in particular, offer an ideal system to investigate which genes and developmental mechanisms contribute to evolutionary diversification. Genetic analyses that try to find the position of genes along chromosomes are invaluable for such efforts, also because they allow researchers to compare chromosome content between species. Here, we report on a study which built a gene-based map for the chromosomes of a butterfly “lab rat” and identified chromosomes carrying color pattern genes. We compare our map to that of the reference lepidopteran species, the silkworm. Despite these species having diverged some 100 million years ago, there is much conservation in terms of which genes are found together in chromosomes and even how genes are ordered within chromosomes. However, because we were able to compare positioning of many more genes than had ever been reported before for this group, we also found evidence of several large- and small-scale chromosomal rearrangements. We discuss the advantages of gene-based maps in understanding the genetic basis of color pattern evolution.

## Introduction

With the need for a wider sampling of biological diversity [1]–[3], the availability of tools for large-scale genetic and genomic analysis is rapidly being extended beyond a handful of classical model systems. Gene collections are growing for various species and with them, the need for methods to assign genes to genetic maps and to assess synteny with relevant sequenced genomes. Gene-based linkage maps are invaluable in the search for the loci that contribute to phenotypic evolution; they are more easily transferable and comparable between species than anonymous markers, and facilitate resolution of mapped genomic regions to candidate genes, also via comparisons of maps or gene functions between species.

The Lepidoptera (butterflies and moths) are a diverse order of insects with an abundance of species, including many agricultural pests, and one of two species of domesticated insects. Lepidopterans have some unusual genetic properties, such as holocentric chromosomes, heterogametic females, and male-restricted meiotic recombination, whose underlying mechanisms and consequences for genome evolution remain to be fully explored. However, lepidopteran species are relatively under-represented in terms of genomic resources with little available outside the model silkworm *Bombyx mori* [4]. Comparative genomics among lepidopterans and a detailed comparative analysis of the *B. mori* genome have been hampered by the relative scarcity of relevant genomic information. Dipterans, the closest insect lineage with available sequenced genomes, diverged from lepidopterans more than 200 MYA, and there is relatively little genomic information within the Lepidoptera. This is especially striking for butterfly species (derived from moths some 100 MYA), despite much interest in their diverse, derived, and ecologically-relevant wing patterns.

Color patterns on butterfly wings include some compelling examples of adaptation and have regained interest in evolutionary developmental biology's quest to understand the mechanistic basis of phenotypic variation [5]–[7]. A number of candidate genes well described in relation to wing development in *Drosophila melanogaster* have been implicated in formation (reviewed in [5],[8]) and variation [9]–[11] of wing patterns in butterflies. Despite the success of the Drosophila-based candidate gene approach, it is clear that a more unbiased approach will be necessary. For example, for those cases where there are no obvious candidate genes [12], and because it is conceivable, if not likely, that genes other than those described for a derived model system will be relevant for traits that are restricted to a lineage diverged more than 200 MYA. For this reason, there have been a number of recent efforts to push forward butterfly genomics [13],[14], including construction of large EST collections [15]–[17], and genetic linkage maps [18]–[21] for a few target species. The latter are, however, largely or exclusively composed of anonymous markers, limiting broad-coverage comparative analysis of gene co-segregation and order across species. Recent studies based on a limited number of pairs of mapped orthologous markers have proposed conservation of syntenic blocks and gene order between *B. mori* and *Manduca sexta* moths and/or *Heliconius melpomene* butterflies [22]–[25]. Extending this type of analysis to many more pairs of mapped orthologs will be crucial to exploring the use of *B. mori* as a pan-lepidopteran genomics reference, and to allow integration of genomics information now accumulating for different species of butterflies [14].

*Bicyclus anynana* is probably the closest to a butterfly equivalent of a “lab rat”. This species was introduced to captivity some two decades ago and it has since been the focus of studies on the evolution and development of wing patterns and other phenotypes [26]. Two key processes in morphological evolution are captured on the wings of these butterflies; diversification of evolutionary novelties (as are the scale-based color patterns of butterflies [27]), and of serially-repeated structures (as are the eyespots of many Nymphalids [28]). Laboratory populations of *B. anynana* have been used to examine the genetic, developmental, and physiological basis of phenotypic variation [29], and have provided the material for identification of anonymous [30] and expressed gene-based [15],[31] markers. Here, we describe a study that genetically maps SNPs in a large number of ESTs to *B. anynana* chromosomes. We use a mapping panel composed of a number of small families to maximize the number of mapped markers and produce the densest gene-based map available to date for any butterfly species. This map includes a number of color pattern loci defined by spontaneous Mendelian mutations and enabled a large-scale analysis of synteny with the lepidopteran reference species. The usefulness of gene-based linkage maps and comparative analysis of chromosomal composition is illustrated in relation to the identification of color pattern loci.

## Results/Discussion

We used a biphasic linkage mapping method [32] to map 508 markers on expressed genes and seven color pattern loci in *B. anynana* linkage groups (LGs). With the largest collection of anchor loci mapped to date for any butterfly species, we were able to do a broad-coverage comparison of gene co-segregation and gene order between *B. anynana* butterflies and the lepidopteran reference species, *Bombyx mori*. Our analysis confirmed previous reports of conserved synteny in the Lepidoptera and also detected several small- and large-scale chromosomal rearrangements separating *B. anynana* butterflies and *B. mori* moths.

### Expressed Gene-Derived and Color Pattern Markers

We selected 768 SNPs in expressed genes to genotype in a mapping panel composed of 288 individuals from 12 F2 families (Table S1). These markers correspond to 745 SNPs in 744 UniGene contigs (marker name starting with BaC) and 23 SNPs in 14 selected candidate genes (marker name starting with BaG). The contigs were identified from the assembly of over 100,000 EST reads, and the candidate genes were selected based on their developmental roles (see Methods). We selected a single SNP for most genes, with the exception of 5 genes whose potential role in development warranted extra effort. Seventy percent (533 of 768) of the target SNPs converted into good assays, defined as those with 90% of the panel individuals being genotyped and with a minor allele frequency greater than 5%. For these SNPs (Table S2), each of the 12 families had an average of 60 SNPs that were informative in females only (ranging from a maximum of 84 to a minimum of 43), 63 SNPs that were informative in males only (ranging from 81 to 44), and 141 SNPs that were doubly-informative (ranging from 155 to 119). On average, each of those SNPs was male-informative only in 1.4 families, female-informative-only in 1.3 families and both male and female informative in 3.2 families. Upon visual inspection of the genotypes for the 533 markers (Table S2), we identified 513 markers with autosomal segregation patterns, 9 with segregation patterns consistent with sex linkage, and 11 with several Mendelian inconsistencies which were excluded from further analysis.

**Figure 1:**
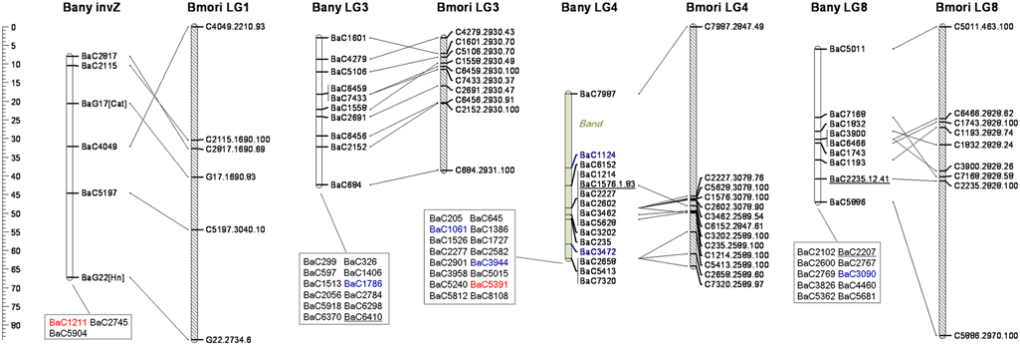
*Bicyclus anynana* linkage map and pan-macrolepidopteran synteny. Orthologous *B. anynana* (empty) and *Bombyx mori* (dashed) LGs are shown with lines connecting orthologous markers. Numbering of *B. anynana* LGs reflects homology with *B. mori* (except for LG28). LGs labeled with “inv” were inverted to match marker order in the other species. Only those *B. mori* markers with mapped *B. anynana* orthologs are shown; their label follows the format A.B.C, with A = corresponding *B. anynana* marker name except for the “Ba” prefix, B = *B. mori* scaffold containing the marker, and C = log10(e-score) of the corresponding blast. Ordered markers are shown along the LGs (cM distances between *B. anynana* markers can be seen on scale to the right of each row of LGs), and unordered markers inside boxes. Marker names are shown in green if they correspond to visible mutations (likely position displayed as green shading inside the corresponding LG), blue if they either do not blast any *B. mori* scaffold or blast a *B. mori* scaffold which is not mapped, red if they blast a *B. mori* scaffold on a non-orthologous (i.e. different-number) *B. mori* LG, and black for all markers in orthologous *B. mori* LGs. Underlined *B. anynana* markers have mapped orthologs in *Heliconius melpomene*; nomenclature D.E.F for ordered markers follows: D = *B. anynana* marker name, E = *H. melpomene* linkage group [23] it blasts to, and F = log10(e-score) of the corresponding blast (same information for unordered markers is available in Table S4).

Absence of recombination in lepidopteran females can be exploited to construct genetic linkage maps via “biphasic mapping” [32]; marker pairs that are female fully-informative are used to initially assign markers to segregation groups, and male informative markers are then used to order markers within those groups. We used this strategy and CRIMAP software for pedigree analysis [33] and were able to assign 508 SNPs to 28 *B. anynana* linkage groups (Table 1; Figures 1–4), possibly corresponding to the 27 autosomes and Z sex chromosome of this species [20]. We were able to map 10 of our 14 candidate genes (BaG markers): *cubitus interruptus* (*ci*), *Ecdysone receptor* (*EcR*), *engrailed* (*en*), *APC-like* (*Apc*), *naked cuticle* (*nkd*), *cinnamon* (*cin*), *Henna* (*Hn*), *echinus* (*ec*), *Catalase* (*Cat*), and *Heat-shock protein 70* (*Hsp70*). Failure to map the other four candidate genes was due to a failed assay (*split ends*, *spen*), Mendelian inconsistencies (*scabrous*, *sca*), or the SNP being fixed in the mapping panel (*wingless*, *wg* and *groucho*, *gro*).

**Figure 2 pgen-1000366-g002:**
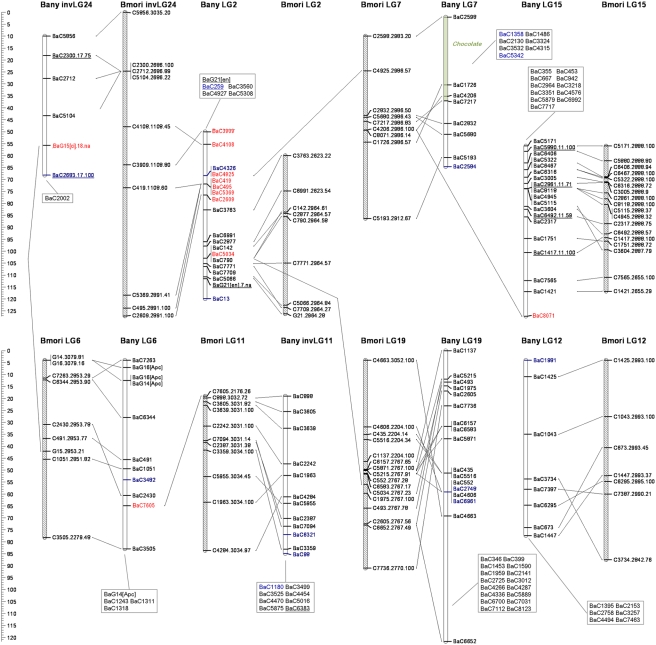
See legend to Figure 1.

**Figure 3 pgen-1000366-g003:**
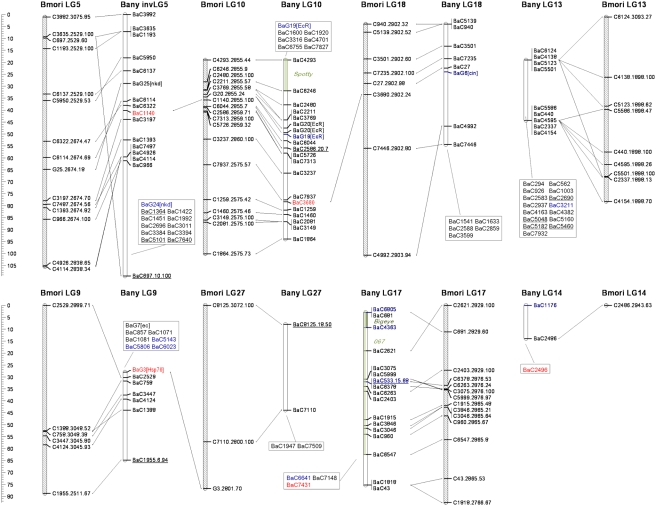
See legend to Figure 1.

**Figure 4 pgen-1000366-g004:**
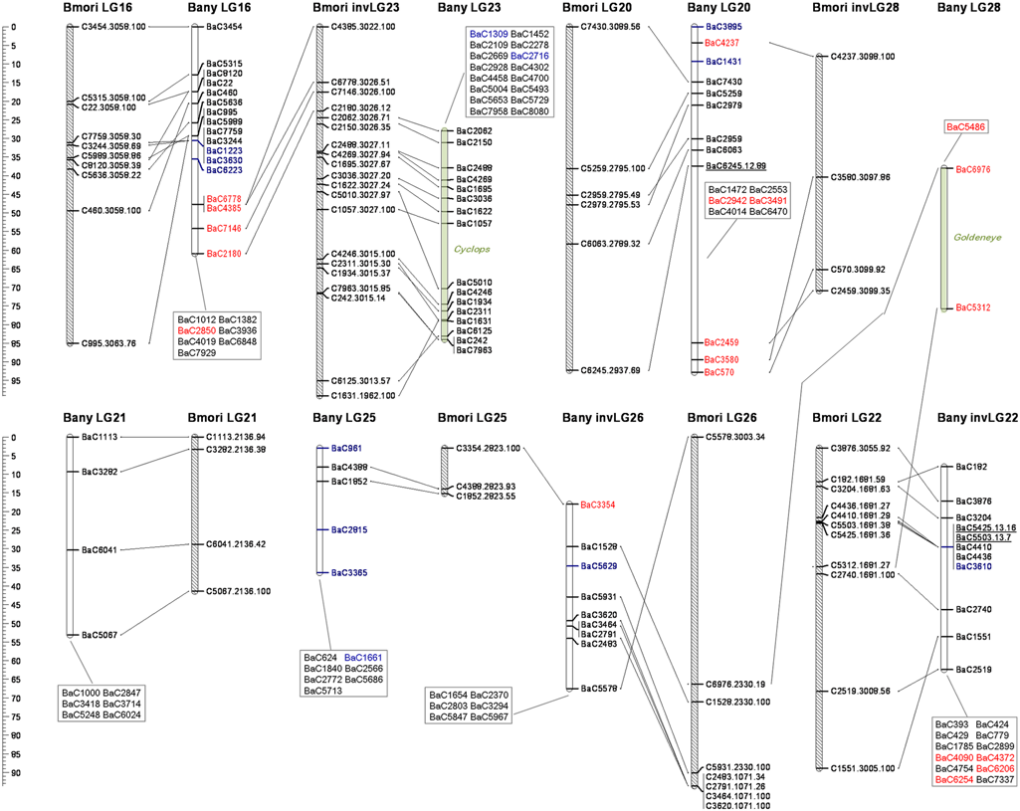
See legend to Figure 1.

**Table 1:** Summary of mapping information for the gene-based markers assigned to *Bicyclus anynana* linkage groups.

Bany	# markers		Female distances			Male distances				% map	# orthologs	
LG	O	U	f cM	int>0	f int	m cM	mean	max	blocks	exp	Bm	Hm
Z	6	3	2.3	1	0.5	59.2	11.8	22.6	0|0	4	9	0
2	19	5	24.3	7	1.4	69.9	3.9	13.3	3|8	35	21	2
3	10	12	1.7	1	0.2	39.4	4.4	10.2	1|2	4	21	1
4	15	16	6.5	2	0.5	44.1	3.2	19.8	2|9	15	27	1
5	16	11	4.6	4	0.3	109.4	7.3	48.2	2|5	4	26	4
6	11	4	15.9	3	1.6	78.9	7. 9	18	1|2	20	14	0
7	8	7	0	0	0	62.6	8.9	28.4	0|0	0	12	0
8	9	10	1.5	2	0.2	41.0	5.1	18.3	1|2	4	18	2
9	7	7	0	0	0	36.8	6.1	20.9	0|0	0	11	1
10	20	7	5.2	5	0.3	74.9	3.9	12.9	3|6	7	25	1
11	12	8	13.5	3	1.2	65.9	6.0	14.8	0|0	20	17	1
12	8	6	40.3	3	5.8	73.4	10.5	24.1	0|0	55	13	0
13	9	15	9.5	1	1.2	25.3	3.2	25.3	2|9	38	23	4
14	2	1	0.2	1	0.2	13.9	13.9	13.9	0|0	1	2	0
15	19	11	8	6	0.4	71.2	4.0	11.7	3|6	11	30	4
16	17	7	7.8	6	0.5	60.9	3.8	12.9	4|10	13	21	0
17	17	3	23.9	6	1.5	72.3	4.5	12.8	4|8	33	16	1
18	8	5	4.7	3	0.7	50.4	7.2	22.7	1|2	9	12	0
19	17	16	14.5	4	0.9	121.7	7.6	52.5	3|8	12	31	0
20	12	6	18.2	4	1.7	92.8	8.4	47.5	0|0	20	16	1
21	4	6	0	0	0	53.1	17.7	22.8	0|0	0	10	0
22	11	12	4.7	4	0.5	54.4	5.4	16.8	1|5	9	22	2
23	16	16	2.5	1	0.2	56	3.7	17.5	1|2	4	30	0
24	6	1	0.9	1	0.2	58.2	11.6	15.6	0|0	2	6	3
25	5	7	0	0	0	33.4	8.4	13	0|0	0	8	0
26	9	6	0.6	1	0.1	49.5	6.2	13.5	1|2	1	14	0
27	2	2	1.1	1	1.1	35.9	35.9	35.9	0|0	3	4	1
28	2	1	0	0	0	37.7	37.7	37.7	0|0	0	3	0

*Bany LG* = *B. anynana* linkage groups whose number reflects homology with *B. mori* LGs (see Figures 1–4); *# markers* = number of ordered (O) and unordered (U) markers. For the ordered markers: *f cM* = female map size; *int>0* = number of cases where “female distance” between consecutive markers is not zero, *f int* = average “female distance” for all consecutive markers; *m cM* = male map size; *mean* = average distance between markers in males (not excluding intervals with distance = 0 cM); *max* = maximum distance between markers; *block* = number of blocks of markers mapping to same cM position | number of markers in those blocks. *% map exp* = map expansion (calculated as *f cM* / *m cM*). For all markers: # *orthologs* = number of markers with mapped orthologs in *Bombyx mori* (Bm) and *Heliconius melpomene* (Hm). All data for ordered and unordered markers are available in Tables S3 and S4, respectively.

We also attempted to map nine Mendelizing mutations affecting body or larval coloration which were segregating in some of the 12 full-sib mapping families (Table 2). Two of the visible markers could not be mapped (LOD score not significant), and the other seven were assigned to six LGs. Mapped markers typically had poorly resolved map positions, often corresponding to the entire length of the chromosome (Figures 1–4, Table 2). Among the mapped visible mutants, two are particularly worrisome: 1) the *Spotty* mutation for which a 2 LOD support interval included positions at either end, but excluded the middle region of LG10 , and 2) the *Goldeneye* mutation which mapped to LG28 whose validity we are uncertain of (see below). Poor mapping resolution for the visible markers likely reflects the fact that: 1) any given mutation was typically only segregating in 1–4 families (Table 2), 2) in the case of non co-dominant mutations a fraction of the segregants needed to be scored as “missing” which resulted in further loss of resolution, and 3) the mutations may not be 100% penetrant. Nonetheless, the mapping of these mutations to chromosomes is a very valuable first step towards efforts to clone the corresponding loci. Fine mapping efforts need now only employ markers in the same linkage groups.

**Table 2:** Visible mutants in mapping panel.

Mutation	Family	Segregation	Phenotype	Ref	LG	Interval
*Bigeye*	1–4	Dominant (homozygous embryonic lethal)	Enlarged eyespots	[51]	17	[BaC6805, BaC4363]
*comet*	1,5,6	Recessive	Comet-shaped eyespots	[60]	NA	NA
*067*	1	Recessive	Enlarged hindwing eyespots 6 and 7	[26]	17	[begin, BaC6547]
*Chocolate*	2,4	Dominant	Darkened cuticle of late-instar larvae	[26]	7	[BaC2598, BaC7217]
*Band*	3	Dominant	Light distal-half of ventral wing surface	[26]	4	[begin, end]
*Spotty*	3	Co-dominant	Extra eyespots on forewing	[51],[52]	10	[begin, BaC6246]
*Cyclops*	3	Dominant (homozygous embryonic lethal)	Vestigial venation and fused eyespots	[51]	23	[begin, end]
*Goldeneye*	5,6	Dominant (homozygous embryonic lethal)	Black scales of eyespot replaced with golden scales	[27],[50]	28	[begin, BaC5312]
*Missing*	5,6	Co-dominant	Reduced hindwing eyespots 3 and 4	[52]	NA	NA

For each visible marker, we list which families were segregating for the mutant allele, and the marker's segregation properties and phenotype plus references where it has been described. We also report on the LG where the marker mapped to, and the one LOD support interval (see also Figures 1–4; NA is used for those markers we were not able to map).

With 508 markers in expressed genes and seven visible mutants, this is the densest non-anonymous marker map ever reported for a butterfly species. Up until now, the most anchor loci mapped in this group was 101 for *Heliconius melpomene* (*cf.* [23]), another Nymphalid.

### *B. anynana* Gene-Based Linkage Map

For all the SNPs assigned to a given segregation group we used male informative markers to build a map for that group (Figures 1–4). For 297 of the 508 gene-based markers, we were able to assign a position in the corresponding LG (hereafter, “ordered markers”; Table S3). The remaining 211 markers were not assigned to a unique position, but their position was typically narrowed to two or three intervals (hereafter, “unordered markers”; Table S4). LGs had on average 10.6 ordered and 7.5 unordered markers with standard deviations of 5.5 and 4.6 respectively (Table 1). Three linkage groups (LG24, LG27, and LG28) consisted only of single ordered markers at the tips and zero to two extra, unordered markers. In addition, despite a total of 24 markers assigned to LG13, this LG only had markers placed at the tips. The reasons for poor marker resolution in LG13 are unknown.

Our total estimated map length, based on LG “male-based” distance between terminal markers, was 1642.2 cM, with individual LG length varying between ∼14 cM (LG14) and ∼122 cM (LG19) (Table 1). This map length is well within that estimated for different butterfly species (1430 cM–2542 cM, [18],[19],[21]) and close to that estimated for *B. anynana* based largely on AFLP markers (1354 cM or 1873 cM depending on the mapping software used *cf.* [20]). However, because of the probable non-zero distance between terminal markers and chromosome ends, the “male distance” between terminal markers can be an underestimate of actual LG lengths.

Our dataset allows for two types of quality control of map assignments. First, estimates of map distance in females (which should be zero since they do not have recombination) is a measure of potential map expansions due to errors. About 26% (70 of 269) of the “female distances” between neighbor ordered markers were greater than 0 cM (Table 1). The average distance for the non-zero distances was 3.0 cM, and included 11 distances greater than 5 cM, and 4 greater than 10 cM (Table S3). The total female map is 212.4 cM implying a map expansion due to genotyping errors and/or the mapping algorithm of ∼12.9%. The extent of this expansion varies greatly between LGs (Table 1); while for most, the expansion is lower than 10%, for LG12 it reaches 55% (due mainly to a single terminal marker; see Table S3). Secondly, male recombinational distance between multiple SNPs at the same gene measures error in map position assignments. We have two genes where multiple markers have been ordered, *Apc* (LG6) and *EcR* (LG10). For *Apc* two of the three ordered markers overlap and the third maps at a distance of 5.4 cM from them, while for *EcR* all three markers map to positions within 2.6 cM from each other (Figures 1–4). The average maximum distance between ordered non-overlapping markers at the same locus is 4 cM, and the average distance of the four possible distances between consecutive markers (0, 5.4, 1.9, 0.7) is 2 cM. Some of this error is certainly associated with genotyping errors, but it may also result from our mapping approach which attempts to integrate marker information over several families (see below). In any case, this analysis suggests that distances smaller than ∼5 cM might not be well resolved in our map.

### Mapping Strategy

Our method was designed to maximize the number of gene-based markers assigned to linkage groups with minimum *de novo* SNP identification. This approach involved: 1) focusing on SNPs identified in EST collections (thus, in expressed genes) and for which the minor allele was seen at least twice (thus making it less likely that SNPs are cDNA-related errors; [31]), 2) using a mapping panel composed of a number of small families rather than one large one (maximizing the number of mapped markers at the expense of their mapping resolution; see below) and CRI-MAP software for pedigree analysis, 3) using Illumina GoldenGate genotyping technology (without any per SNP assay optimization), and 4) following a biphasic linkage mapping method [32] which takes advantage of the fact that there is no recombination in lepidopteran females.

We chose to use a mapping panel made up of a number of small families rather than the more typical single large family. With this strategy we maximize the chance of assigning any given SNP to a LG (as this only requires having one female informative family), and, once assigned to a linkage group, we maximize the chance of identifying at least a second family in which that SNP is (also) male informative. Of the 508 mapped markers, 11 corresponding to nine Z-linked loci and to two autosomal markers (BaC645 on LG2 and BaC4454 on LG11; *cf.* Methods) were not female-informative (i.e. heterozygous) in any family. Similarly, only three SNPs were not male informative in any family (Table S2). A panel derived from several independent parental pairs, additionally allows for estimates of population SNP frequencies, which will be useful in future mapping experiments. The disadvantages of this strategy are noticeable in terms of mapping resolution when a marker is male informative only in a single (or few) families and because of the need to integrate marker information across families. The majority of the SNPs (264 of 508) were male-informative (including both SNPs informative only in males and those doubly-informative) in at least five families (corresponding to at least 120 individuals in the mapping panel), and three SNPs were informative for a maximum of ten families (240 individuals).

In downstream uses of this map (*e.g.*, for mapping QTLs or visible mutants), we will be able to choose from mapped, intermediate-frequency, informative SNPs, to design assays for larger mapping panels derived from a smaller number of founders. For this, having a large number of gene-based markers (even if mapped with limited resolution) and knowledge of SNP frequency is more useful than having a very accurate map of sparse markers (which may not be informative in another context).

### Pan-Lepidopteran Reference Genome: The Silkworm *Bombyx mori*

A recent very high density SNP map for *Bombyx mori* [34] combined with a new assembly (unpublished) of the whole-genome sequence of this species [35],^36^ with larger average scaffold sizes, may be used as a pan-Lepidoptera reference. Using blastn, we assigned 1711 of the 1755 mapped SNPs in the silkworm *B. mori* [34] to the recent genome-sequencing scaffolds [37] (Table S5). The mapped markers aligned to 185 of the 645 different scaffolds, consistent with the highly skewed distribution of scaffold lengths. Of the 185 scaffolds to which markers mapped 29%, 10%, 6%, and 6% had one through four mapped markers, respectively. On the other hand, ∼90% of the mapped markers were assigned to only 91 scaffolds having more than four markers each, implying that the bulk of the current *B. mori* genome assembly is contained in 91 large scaffolds. As a check on the quality of the current assembly, we looked for scaffolds with more than four mapped markers in which at least one marker mapped to a different linkage group than the remainder. We observed seven scaffolds (7.7%) with material coming from two chromosomes and two additional scaffolds (2.2%) with material derived from three chromosomes, suggesting that there are errors with the current assembly. A visual inspection suggests that those assembly errors tend to be associated with the very ends of scaffolds. So, although the fraction of large scaffold with such errors is significant, very little of the assembly is affected. We next fitted local regressions for each scaffold that allowed for predictions of genetic positions (cM) given a physical position (bp) on the scaffold (see Methods). The *B. mori* map thus generated was the basis for the comparative analysis with our *B. anynana* gene-based map.

The current *B. mori* map consists of over 1650 SNPs covering 1413 cM [34]. With 28 chromosome pairs, *B. mori* has the largest chromosome number of all insect genomes sequenced to date. Previous studies of deep synteny across insects showed that divergent gene order correlates with divergent protein sequence [38], and that there is more conservation of syntenic groups between *B. mori* and the coleopteran *Tribolium castaneum* than between *B. mori* and the hymenopteran *Apis mellifera* [34]. Both these orders have presumably split from a common ancestor with lepidopterans earlier than dipterans did. However, analysis of synteny blocks with sequenced representatives of the Diptera is hampered by the large difference in chromosome number; typically around 30 pairs in most lepidopteran species [39] and between three and six pairs for the various sequenced dipteran (mosquito and Drosophila) species. Among lepidopterans, and despite the available phylogenetic framework for comparative analysis (e.g. [40]–[44]), relatively sparse genomic resources have resulted in very few attempts to examine synteny. Previous studies compared synteny blocks between moths and *Heliconius* butterflies based on a modest number of mapped orthologous pairs (maximum 72 with many “unordered” [23]). Here, in a comparison between *B. mori* and *B. anynana* genetic maps, we increased this number by more than seven times, with a large fraction of our markers being “ordered”. This type of analysis, hopefully extending also to representatives of the microlepidoptera (all lepidopterans examined to date are macrolepidopterans), will be crucial to put the gene collections and genetic maps, growing for a variety of butterfly species, into phylogenomic context.

### Pan-Macrolepidopteran Macro-Synteny: Conservation of Chromosomal Gene Composition

We used blast to identify orthologs of the gene-based markers in *B. anynana* (Neolepidoptera; Papilionoidea; Nymphalidae; Satyrinae) mapped in other lepidopteran species: the butterfly *Heliconius melpomene* (Neolepidoptera; Papilionoidea; Nymphalidae; Heliconiinae), and the silworm *Bombyx mori* (Neolepidoptera; Bombycoidea; Bombycidae; Bombycinae). Of the 508 *B. anynana* markers, 29 (18 ordered and 11 unordered) had an ortholog among the 101 anchor loci mapped in *H. melpomene* [23], and 462 (269 ordered and 193 unordered) could be assigned to a mapped *B. mori* scaffold. Of the remaining 46 mapped *B. anynana* markers (blue in Figures 1–4), 20 had orthologs in *B. mori* scaffolds which we could not assign to a *B. mori* LG and 26 did not have significant sequence similarity with any *B. mori* scaffold.

**Figure 5:**
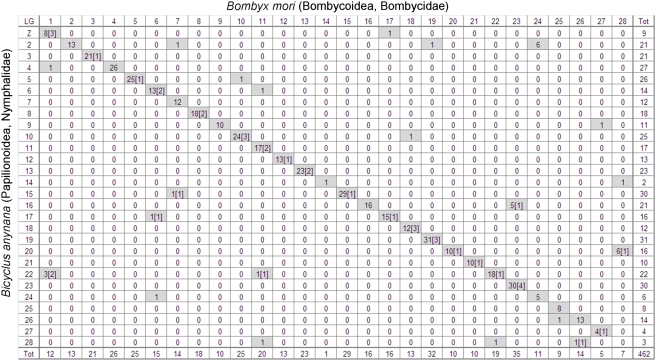
Oxford Grid representing conservation of marker co-segregation between *Bicyclus anynana* and *Bombyx mori* linkage groups. For each inter-specific LG pair, the number in the cell represents the total number of mapped orthologous markers (blast cut-off e-score 1.0e-05; numbers in brackets correspond to number of blast hits with e-scores greater that 1.0e-20). Grey shading highlights LG pairs sharing at least one orthologous gene.

Despite the ca. 100 MY that separate butterflies and moths [23],[42], there is much conservation of the grouping of genes in linkage groups (Figures 1–5). Our numbering of *B. anynana* LGs reflects homology with *B. mori* with the exception of *B. anynana* LG28, which has only two markers and none with orthologs mapping to *B. mori* LG28 (Figures 1–4). Of the 462 pairs of mapped orthologous markers in the two species, 425 (∼92%) are found in orthologous LGs (Figure 5). The 37 orthologous genes found on non-orthologous LGs, include 17 that are associated with three large chromosomal rearrangements (involving LG2 and LG24, LG16 and LG23, and LG20 and LG28), and 20 which are potential single gene transpositions. The latter may also include blast false positive (even though only five cases had e-values higher than 1.0e-20; Figure 5), blasts to pseudo- or duplicate genes, or mapping errors (*e.g.*, markers mapping to non-syntenic linkage groups that are isolated at the tips of chromosomes are especially suspicious). Both *B. mori* and *B. anynana* have 28 pairs of chromosomes, while basal lepidopterans have 31 pairs [45] and different species of butterflies and moths have very variable numbers [39], [45]–[47]. The instances where individual *B. anynana* LGs are made up of syntenic blocks from different *B. mori* LGs suggest that the two lineages have undergone independent karyotype reductions, via non-homologous chromosomal fusions.

A previous study compared linkage group assignment for 72 orthologous pairs of markers available for another Nymphalid butterfly (*Heliconius melpomene*) and the reference lepidopteran (*Bombyx mori*) and concluded that extensive synteny existed [22],[23]. Some striking differences, however, are apparent between the genome-wide analysis of macro-synteny for *B. mori* and *B. anynana* (this paper) and that for *B. mori* and *H. melpomene* [23]. First, the comparison between *H. melpomene* and *B. mori* did not detect any of the chromosomal rearrangements we document (Figures 1–4). This may be because these rearrangements are not present in *Heliconius*, or because they could not be detected given the relatively small number of mapped orthologs pairs in *H. melpomene* and *B. mori*. Thus, it remains unclear to what extent the rearrangements we see in *B. anynana* are characteristic of Nymphalid butterflies or more lineage-restricted. Second, we see no evidence of the six chromosomal fusions proposed to distinguish the *H. melpomene* and *B. mori* genetic maps [23]. This probably reflects the fact that *Heliconius* butterflies have a lower chromosome number (21 pairs instead of the 28 pairs in both *B. anynana* and *B. mori*), and must thus have undergone further, or independent, chromosomal fusions relative to *B. anynana*. It is, however, noteworthy that the proposed fusions separating *H. melpomene* and *B. mori* are based on few pairs of mapped orthologous markers (mostly 1–3 pairs [23]) and our analysis shows that single marker transpositions do occur (Figures 1–5).

### Pan-Macrolepidopteran Micro-Synteny: Conservation of Gene Order

Figures 1–4 illustrate synteny between *B. anynana* and *B. mori* orthologous markers: both in terms of the grouping of markers in LGs (see also Figure 5), and in terms of conservation of gene order along individual LGs. For most LGs with multiple ordered markers, we have evidence for some reordering of genes which suggests multiple inversions separating *B. anynana* and *B. mori*.

From the 23 *B. anynana* LGs with greater than three ordered and non-overlapping markers (i.e. excluding multiple markers mapping to the same genetic position), with a mapped *B. mori* ortholog on a syntenic block, only LG10 and LG21 have fully conserved marker order (Figures 1–4). For the remaining LGs, we see evidence of order rearrangements ranging from one (e.g. LG9, LG18) to multiple (e.g. LG17, LG19) markers whose relative position in *B. anynana* differs from that in *B. mori*. Where the marker order inferred for *B. anynana* differed from that of the orthologous markers in *B. mori*, we compared the log10 likelihoods of the two (Table S6). Of the 24 comparisons made (complete LGs or LG fragments with homology to different *B. mori* LGs), inferred marker order in *B. anynana* was at least twice as likely than *B. mori* order in 20 cases (and at least 630 times more likely for 18 of the comparisons). For the four situations where the *B. mori* order was better supported than the one originally inferred for *B. anynana* (LG2, LG6, LG10, and LG17), and for LG13 (which had many but very poorly resolved markers and where the original inferred order was only ∼2 times better than that of *B. mori*), we used the *B. mori* order as a starting point in CRI-MAP and further improved it (see Methods). In all cases except LG10, and the LG2 segment homologous to *B. mori* LG2, the final order was different from that in *B. mori*. The difference between the log10 likelihoods for the final inferred marker order in *B. anynana* and that in *B. mori* ranges between 1.1 for LG13 (*i.e.* the inferred *B. anynana* order is ∼13 times more likely than that in *B. mori*) and 34.8 for LG11 (*i.e.* inferred order ∼10∧34 times better) (Table S6). Because *de novo* map construction using CRI-MAP uses a “hill climbing” algorithm to maximize marker order likelihood, the map order arrived at is dependent on the particular subset of markers used to initiate a build. This explains why the build corresponding to some *B. anynana* LGs reached a local maximum that could be improved upon by using the *B. mori* gene order as seed. Note that marker mapping was further improved by re-adding to the map markers with no mapped *B. mori* ortholog and by re-assessing unordered markers in those LGs. The mapping information in all Tables and Figures corresponds to the final CRI-MAP builds.

Our data suggest that previous conclusions of highly conserved gene order between *H. melpomene* and *B. mori* [23]–[25] may have been over-stated, perhaps as a result of the limited number of markers examined (maximum of 10 ordered orthologous pairs in one syntenic block [25]). Future work adding extra markers and improving marker mapping resolution in *B. anynana*, and extending comparative analysis to additional species will be crucial to rigorously quantify the extent of inversions separating different lepidopteran lineages. Unfortunately, the number of shared ordered markers in the *H. melpomene* and *B. anynana* maps prevents evaluating the consistency of gene order within Nymphalid butterflies. We have a single *B. anynana* LG (LG15) with greater than two ordered markers with mapped orthologs in *H. melpomene*. However, of those four markers, only one has a resolved genetic map position in *H. melpomene* [23] making impossible the assessment of conservation of gene order.

Previous studies that analyzed order of more than three ordered *H. melpomene* - *B. mori* marker pairs were much more localized than the study presented here. They either focused on one individual chromosome (and reported on four perfectly aligned markers [24]), or on a BAC-level scale (and reported on nine of ten aligned markers [25]). However, conservation of gene order for small collections of orthologous markers can occur by chance alone (*e.g.*, four perfectly aligned markers occur by chance ∼10% of the time), and comparisons of marker order at the level of single BACs can only infer conservation at sub-centimorgan scales. Here, we extended the analysis of gene order to many more markers in many more linkage groups and alert for the fact that, even though we have syntenic blocks and broad conservation of gene order (see, for example, LG10), we also have clear evidence of multiple rearrangements (see, for example, LG19). These intra-chromosomal rearrangements do not mean that *B. mori* cannot serve as a pan-macrolepidopteran reference, but they do argue that marker order is likely conserved over tens of centimorgans as opposed to entire linkage groups. Our observations are remarkably similar to the emerging consensus view in the Drosophila clade (including species diverged some 40 MYA), that the assignment of genes to Mullerian elements is highly conserved but gene order within those elements is variable [48],[49]. It will be interesting to look both more widely (across species from different families) and also more narrowly (across multiple species within some selected genera) in the Lepidoptera. It is still unclear how the relatively numerous and relatively small (in insect terms) chromosomes in this diverse group have evolved and what the role of the holocentric chromosome structure and male-restricted recombination has been in the process.

### Mapping Butterfly Color Pattern Loci

**Figure 6:**
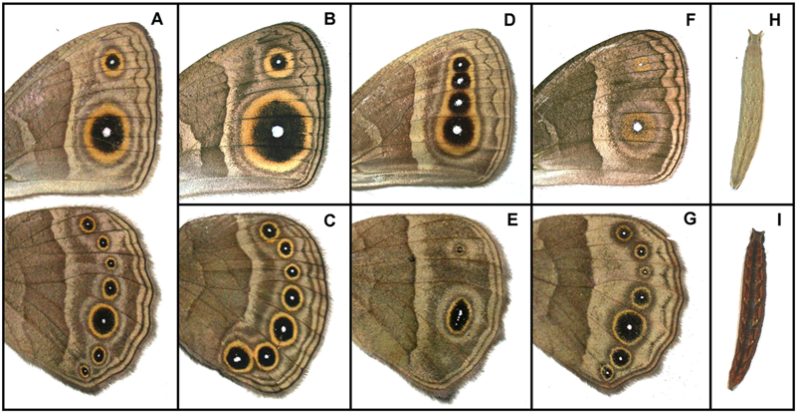
Mapped *B. anynana* pigmentation mutants. Ventral surface of fore- (top) and hind-wing (bottom) of adult butterflies from different laboratory stocks: (A) “wildtype”, and mutants (B) *Bigeye* with all eyespots enlarged, (C) *067* with hindwing eyespots 6 and 7 enlarged, (D) *Spotty* with two extra eyespots on forewing, (E) *Cyclops* with fused eyespots, (F) *Goldeneye* with golden scales replacing the typically black scales of the eyespot mid-ring, and (G) *Band* with lighter distal wing half. Top view of a fifth and final instar larvae of different laboratory stocks: (H) “wildtype”, and (I) *Chocolate* mutant with dark-brown integument.

Aside from enabling analysis of macro- and micro-synteny, gene-based maps are of great value in studies attempting to map genes that contribute to phenotypic variation because they greatly facilitate the resolution of mapped genomic regions into a tractable number of candidate genes. This is not only because the mapping analysis itself can exclude candidate genes (namely, those in non-implicated LGs), and identify candidate genes among available markers, but also because conservation of gene grouping and gene order in related species with dense linkage maps might allow identification of extra candidate genes within the implicated genomic regions. For example, the *B. mori* ortholog to the pigmentation gene *black* localizes to a *B. mori* scaffold (nscaf2986 in [37]) mapping to the *Chocolate*-containing region of *B. anynana* LG7. This makes *black* an interesting candidate gene for the *Chocolate* larval phenotype (Figure 6I).

With the exception of *Bigeye* and *Chocolate* (and the more dubiously mapped *Spotty*; see above), at present we have only mapped our collection of *B anynana* visible mutants to entire linkage groups (Table 2). While this renders identification of individual candidate genes premature, our analysis enables us to clearly identify “anti-candidates”. For example, from a developmental point of view, the gene *engrailed* would be a good candidate for several of our Mendelian mutations. The expression of *engrailed* is regulated in relation to different stages of eyespot development [50], and to changes in eyespot size [29], color-composition [50] and number [51],[52]. The involvement of *engrailed* in eyespot formation and also in embryonic development [27] makes it a potential candidate gene for mutations such as *Goldeneye* and *Bigeye* which affect both embryonic viability and eyespot morphology (Figure 6). However, none of the mapped visible markers maps to the *engrailed*-containing LG2, and hence none can be alleles at this locus. This, of course, does not mean that the *engrailed* locus cannot contribute to complex naturally occurring segregating variation or other laboratory mutations affecting wing patterns. Future studies trying to refine the location of each of our mapped color pattern loci will need only to concentrate on markers throughout single LGs, greatly reducing the genotyping effort.

Another exciting aspect of having color pattern loci in gene-based maps of different lepidopteran species is the possibility to investigate to what extent color pattern diversification in different lineages has a similar genetic basis. Recent studies have shown that color pattern loci contributing to race variation map to homologous genomic regions in different *Heliconius* species [12],[53]. Whether these loci play a role in color pattern variation outside *Heliconius* and to what extent color pattern diversification has repeatedly recruited the same loci in different lineages are interesting questions in evolutionary (developmental) biology. We looked for *H. melpomene* and *B. mori* color pattern loci mapping to orthologous LGs to those where we mapped visible markers in *B. anynana* (Table 3). Particularly interesting is the case of the *B. anynana Bigeye* and *067* spontaneous mutations, both affecting eyespot size (Figure 6, Table 2). We mapped these to LG17, which, based on comparisons to *B. mori*, we know is orthologous to *H. melpomene* LG15 (Table 3). This is the linkage group carrying the color pattern loci above-mentioned which have been implicated in the race-divergence in three different *Heliconius* species [6],[12],[23]. Also, the *Band* mutant with lighter background coloration on the distal section of the wings maps to LG4 whose *Heliconius* ortholog carries a white/yellow color switch locus [10]. In the future, emerging comparative maps in *Heliconius* and *Bicyclus* can be exploited to accelerate the dissection of the genetic basis of wing pattern variation in butterflies; potentially aided by patterns of conserved microsynteny detected for “developmental genes” in insect genomes [54].

**Table 3:** Color pattern loci in orthologous lepidopteran LGs.

	*B. anynana*	*H. melpomene* *	*B. mori* *
LG4	*Band*	*K*	*mst*, *L*, *l-w*, *Spc*
LG7	*Chocolate*	–	*q*, *Gb*, *obt*
LG10	*Spotty*	–	*w-1*, *w-2*, *w-3*, *w-5*, *Dus*, *Dp*
LG17	*Bigeye*, *067*	*Yb/Sb/N*	*B*, *Ws*, *bts*, *ow*
LG23	*Cyclops*	*B/D*	*op*
LG28	*Goldeneye*	–	–

*: References for loci nomenclature and phenotype: *H. melpomene* [12],[23] [25], and *B. mori* [61].

### Concluding Remarks

With gene collections growing for a variety of species, so is the need for methods that enable the mapping of markers in those genes and comparisons with genetic maps of relevant reference species. These maps will aid in the genetic dissection of phenotypic variation in non-model systems, enable analysis of synteny and genome evolution, and facilitate future sequence-assembly efforts. Here, we report on the mapping of 508 markers in expressed genes and seven color pattern loci in an emerging butterfly model system. We used our map to compare gene grouping and gene order with the lepidopteran reference genome. Based on 462 pairs of orthologous markers mapped in *Bicyclus anynana* and *Bombyx mori*, we show that there is extensive conservation of syntenic blocks and gene order but not as much as had been previously suggested. We illustrate how gene-based maps and synteny with relevant species in relation to dissecting the genetic basis of wing pattern variation.

## Materials and Methods

### Butterfly Material

We used different *Bicyclus anynana* laboratory populations to establish a mapping panel of 288 individuals from 12 families. These were all F2 families composed of a F1 mother and father, and 22 offspring (typically 11 females and 11 males). The F2 families were obtained by using single-pairs of P grand-parents that were either from “outbred”, or 1–3 generation inbred (*i.e.*, single brother-sister mating pairs) populations. DNA from thorax or head of freshly frozen butterflies (killed in liquid nitrogen and stored at −80°C until processed) was extracted using the QIAGEN tissue kit following manufacturer's recommendations. Genomic DNA was checked for quality and yield on agarose gel and NanoDrop spectrophotometer. From each of the 288 individuals in the mapping panel, 1.7 µg of genomic DNA in 100 µl of QIAGEN kit elution buffer was dried down (SpeedVac), re-suspended in 20 µl water, and sent to Southern California Genotyping Consortium - Illumina Genotyping Core Laboratory at UCLA [55].

### SNP Markers

We selected 768 SNPs to genotype using the Illumina Golden Gate platform [56]. The 768 target SNPs were identified in 759 expressed *B. anynana* genes (Table S1). These correspond to 744 contigs resulting from the assembly of an on-going, large-scale EST project (sequencing of the new ∼91,000 ESTs (GenBank GE654128–GE745563), assembly of those together with the previously published collection of ∼10,000 ESTs [15] and 13 genes available on GenBank nr database, and discovery and characterization of SNPs will be described elsewhere), and 14 candidate genes identified in previous sequencing efforts (including [15],[29],[57] ). The contig-derived markers (name starting with BaC) correspond to SNPs with a minor allele count of two or greater identified in CAP3 alignments of at least 4 EST reads. We identified ∼1,200 contigs with at least one such “double-hit” SNP and chose the 745 target SNPs based on criteria listed below. The candidate genes (marker designation starting with BaG) were selected based on their potential role in wing color patterns or other phenotypes of interest. The genes *ci*, *EcR*, *en*, and *wg*, as well as others from the Wingless signaling pathway, *Apc*, *gro*, *nkd*, and *spen*, are presumably involved in butterfly wing pattern formation [8],[58]. The genes *cin* and *Hn* are involved in pigmentation. Other candidate genes represent various key biological processes, such as wing disc development (*sca*), programmed cell death (*ec*), lifespan (*Cat*), and stress response (*Hsp70*).

We attempted to choose only one high quality SNP for each gene but, in the case of a minority of putative *B. anynana* homologs of developmental candidate genes, we designed two or more assays. These were: two SNPs in the pigmentation gene *yellow* (BaC4163), in *ci* (BaG15), *en* (BaG21), and *nkd* (BaG24 and BaG25); and four SNPs in *Apc* (BaG14 and BaG16), and *EcR* (BaG19 and BaG20). Many criteria went into choosing the target SNPs, including: the estimated frequency of the SNP (preference given to SNPs with high frequency of the minor allele), absence of secondary polymorphisms in the ∼100 bp up- and down-stream of it, the contig annotation (preference given to markers in genes with sequence similarity to genes in public databases), and score for Illumina “type-ability”. Sequences associated with the 768 markers we attempted to genotype are available in Genbank's sequence or EST archive; accession numbers in Table S1.

### Initial Data Filtering

A large fraction of the SNPs assayed converted into working assays and ∼75% had a call rate of greater than 95%. The poorest 15% of SNP assays had a call rate of 0%, whereas the best 80% had a minimum call rate of 89%. The individuals in our genotyping panel consistently generated good data; a 95% Confidence Interval on the number of called SNPs over individuals was 626 to 644 with the poorest and second poorest individuals yielding 527 and 600 called SNPs, respectively. Consistent with this narrow confidence interval, we did not consider removing any individuals from the study because of poor quality DNA. The vast majority of SNPs were ascertained from an EST project so the observation that 15–25% of the attempted SNPs did not convert to a useful assay was not unexpected. Reasons for failure to convert include factors such as: SNPs having a low allele frequency in the mapping panel, some SNPs being falsely identified due to over assembly problems, introns resulting in non-functioning Golden-Gate assays, and errors in flanking regions that the Golden-Gate oligonucleotides anneal to [31]. We chose to focus solely on SNPs for which greater than 90% of the genotyped individuals were “called” and whose minor allele frequency over all called individuals was greater than 5%. These criteria resulted in a set of 533 “converting” SNPs (∼70% of assays attempted). Most SNPs not meeting our inclusion criteria were very clearly failed assays, so either relaxing or increasing the stringency for a SNP's inclusion did not greatly change the number of SNPs in further analyses.

### Manual Curation

We visually examined the dataset for clear genotyping errors that resulted in a SNP showing a pattern of inheritance inconsistent with Mendelian expectations (Table S2). SNPs fell into three categories: 1) inheritance that was sex linked (nine SNPs), 2) several Mendelian inconsistencies (eleven SNPs), or 3) no or a handful of Mendelian inconsistencies (513 SNPs). Sex-linked SNPs were duly noted as they were treated differently in subsequent steps, and SNPs showing several Mendelian inconsistencies (possibly genotyping mistakes, duplicated genes, gene families) were excluded from further consideration. For the SNPs with no or a small number of Mendelian inconsistencies, we manually changed the genotypes of those inconsistent individuals to missing. In the majority of cases this meant discarding the genotype of 1–2 of the 22 full-sib offspring in a family, but in a minority of cases the most parsimonious change involved discarding a parental genotype. This set of 513 hand-annotated putative autosomal SNPs plus the nine sex-linked SNPs were used in all subsequent mapping analysis.

### Assigning Markers to Segregation Groups

We used marker-pairs that were female fully-informative (e.g., dad = aabb & mom = AaBb) to initially assign markers to segregation groups. For all possible pairs of SNPs in the 513 putative autosomal SNPs, we calculated a LOD score summarizing the evidence for complete linkage (LOG10[L(data;r = 0)/L(data;r = 0.5)]). For any given pair of SNPs that LOD score could be missing (if that pair of SNPs was never female fully-informative across the 12 families) or summarize linkage information from 1 to 12 female fully-informative families. We then grouped SNPs connected by LOD scores of greater than eleven. As a result, a SNP could be assigned to a segregation group despite not having a LOD score of greater than 11 with *all* the SNPs in that group. Unpublished simulations suggested that this algorithm rarely results in “over-clustering”. At our CRIMAP inclusion LOD score of 11 we identified 27 segregation groups, with the smallest number of markers in any given group being three, the largest 28 and the mean 13.6 SNPs. Increasing the LOD score for inclusion to values as high as 16 resulted in fewer SNPs assigned to segregation groups, and never split a segregation group identified at an inclusion value of 11 into two. Whereas decreasing the LOD score resulted in the merging of segregation groups (and fewer than 27 clusters).

### Ordering Markers within Segregation Groups

For all the SNPs assigned to a given segregation group we used CRIMAP [33] to build a map for that group. As a result of our having 12 full-sib families, in many instances in which there existed a female informative SNP in one family, at least one other family displayed a: 1) male fully-informative SNP-pair (e.g., dad = AaBb & mom = aabb), 2) a male semi-informative SNP-pair (e.g., dad = AaBb & mom = Aabb), or 3) a doubly-informative SNP-pair (e.g., dad = AaBb & mom = AaBb). CRIMAP was designed for integrating such information in complex human pedigree data [33]. We wrote scripts to take the genotyping data for all the SNPs within a segregation group, irrespective of inheritance patterns, and create input files for CRIMAP. We then used the “build” option of CRIMAP to make a consensus map for each segregation group using default parameters, with the exception of lowering the PUK_LIKE_TOL from 3.0 to 1.0. We built a sex-chromosome map using CRIMAP by simply encoding the “second-allele” in each female as a “9” (i.e., an allele not present).

We manually inspected the resulting maps. In cases where the two “ordered-loci” used to initialize the Expectation Maximization algorithm underlying CRIMAP were loosely linked we re-ran the build option with a different set of random starting loci. In other cases where we observed SNPs that were completely linked in males we re-ran the “build” using the “hap_sys” option for those SNPs. We then used the “flips4” option on the ordered loci to confirm that our maps had the highest possible likelihood, creating a new order when necessary, and re-running the “build” and “flips4” analysis until the order stabilized.

We then used the “two-point” option in CRIMAP in an attempt to assign to segregation groups the remaining 146 putative autosomal markers, not initially assigned. This resulted in our being able to: 1) assign 134 markers to pre-existing segregation groups, 2) merge two pairs of pre-existing linkage groups in single groups, and 3) identify two novel small linkage groups (one having three and the other four SNPs). Typically, added markers displayed a high LOD score for linkage with several members of a pre-existing linkage group and below background LOD scores with members of any other group. The few SNPs that could not be assigned to any segregation group were typically only informative in a single family and/or showed segregation patterns that were unlikely under Mendelian inheritance. Based on the newly defined segregation groups and starting with the markers ordered in the previous round, we carried out another round of “builds”, followed by another round of “flips4”, and iterating until we achieved an ordering for which the “flips4” command could no longer identify orders with higher likelihoods. Details about the mapping of the ordered and unordered markers are found in Tables S3 and S4, respectively.

### Mapping of Visible Mutants

A total of nine Mendelizing visible mutants affecting adult or larval coloration were segregating in six of the twelve full-sibs families used for mapping (Table 2). All offspring of these families were phenotyped and 22 were selected so that each phenotypic class was represented in approximately similar numbers in the mapping panel. Consequently, segregation patterns of the visible mutants in the mapping families do not follow Mendelian ratios. To assign each visible marker to a linkage group, we used the “two-point” option of CRIMAP. This allowed us to assign seven of the nine mutant genes to linkage groups. Despite attempts with lower LOD threshold scores and/or examining only a subset of families we were unable to assign the other two mutants, *comet* and *Missing*, to linkage groups. For the seven mutants mapping to linkage groups we used the “all” option of CRIMAP separately for each mutant and its respective linkage group in an attempt to localize that mutation within a linkage group.

### Orthologous Markers in Other Lepidopteran Species

For all genes in the *B. anynana* map, we used blastn and tblastx analysis (e-score cut-off value of 1.0e-05) against the scaffolds from the *B. mori* genome assembly (May 1, 2008; only the “nscaf” fasta entries from [37]), and against the mapped anchor loci in *Heliconius melpomene* [23]. Two genes in our collection (*ci* and *en*) did not have a significant direct blast hit to any of the target *H. melpomene* markers (“na” notation in marker name in Figures 1–4). However, we were able to identify orthologous pairs based on annotation available for both species via blast analysis to collections from other species. For the contigs with a *B. mori* ortholog, we used custom prediction algorithms (see below) to estimate its position in the *B. mori* map. Details of the blast analysis with *B. mori* and *H. melpomene* can be found in Tables S3 and S4 for the *B. anynana* ordered and unordered markers, respectively.

### Integration with a *Bombyx mori* Map

We downloaded the new collection of *B. mori* scaffolds and used blastn to query all the mapped *B. mori* SNPs from (“DE” accessions from [34]) against the collection [37] (Table S5). We then wished to develop a prediction equation for every scaffold, that when given a base position on that scaffold would return the map position associated with that base position. Such a prediction equation would allow us to estimate a *B. mori* map position for any *B. mori* gene. For *B. mori* scaffolds having greater than four mapped markers this predictor is simply the slope and intercept obtained from a linear regression of map position on base position (of the midpoint of the highest scoring blast hit). For *B. mori* scaffolds with one to four markers this predictor is simply the average position of the markers mapping to that scaffold. For scaffolds with no mapped markers the predictor is undefined. This heuristic seemed reasonable, as a large fraction of the genome is contained in scaffolds with more than four mapped markers, and scaffolds harboring four or fewer markers are typically small enough that returning a single map position for the midpoint of that scaffold is acceptable. During this annotation effort we discovered a small number of *B. mori* scaffolds with termini mapping to different chromosomes, we assumed these are mis-assembly errors and removed these sections of scaffold from further consideration.

### Assessing Differences in Marker Order Relative to *Bombyx mori*

We wished to ask if within linkage group, inferred marker orders in *B. anynana* were different from those in *B. mori*. To do this we used the “fixed” option of CRIMAP to compare the inferred (non-haplotype system) order in *B. anynana* to that in *B. mori* for the subset of markers having orthologs. This analysis allowed us to obtain log10 likelihoods for both orders, and identify instances where the *B. mori* order was more highly supported (Table S6; see Text S1 for an explanation of the contents of all supplementary tables). In those cases we used the *B. mori* order as a new starting point, incorporated any observed haplotype systems, and reran the “flips” analysis to look for iterative improvements over the *B. mori* order. In cases where the “flips” analysis improved upon the *B. mori* order we obtained a log10 likelihood indicating the support for this new order over the *B. mori* order. We then used the order resulting from the “flips” analysis as a seed for an additional “build” run (to possibly place additional unordered markers and/or previously ordered markers without a *B. mori* ortholog). This final build went though additional “flips” rounds and then “fixed” was run on the final order to obtain the map displayed in Figures 1–4.

### Graphical Maps

We used the MapChart software [59] to build a graphical representation of the *B. anynana* genetic map, and of synteny between *B. anynana* and *B. mori* chromosomes (Figures 1–4) . The map produced by MapChart was further processed to include unordered markers and visible mutations. *B. mori* markers were named with the corresponding *B. anynana* marker name, *B. mori* scaffold number, and blast e-score (see legend to Figures 1–4). For the graphical display of the synteny analysis, we multiplied estimated map positions of *B. mori* markers by a factor of two so as to facilitate visualization of homologies with the otherwise relatively condensed *B. mori* LGs. For markers with an estimated position of less than 0 cM, that marker's position was set as 0 cM and the positions of other markers on same linkage group were adjusted accordingly.

## Supporting Information

Table S1 Information on the 768 target SNPs (including frequency, sequence and conversion).
(0.54 MB TXT)

Table S2 Genotypes of 288 panel individuals for each of the 533 converted SNPs.
(1.04 MB XLS)

Table S3 Mapping details for ordered markers including comparisons to *B. mori* and *H. melpomene*.
(0.03 MB TXT)

Table S4 Mapping details for unordered markers including comparisons to *B. mori* and *H. melpomene*.
(0.01 MB TXT)

Table S5 Location of mapped *B. mori* markers in the unpublished scaffold sequences of *B. mori*.
(0.07 MB TXT)

Table S6 Statistical comparison of marker order inferred for *B. anynana* and that in *B. mori*.
(0.01 MB TXT)

Text S1 Readme: text file explaining contents of each of the supplemental tables.
(0.01 MB TXT)
